# Expression of a RhoA-Specific Guanine Nucleotide Exchange Factor, p190RhoGEF, in Mouse Macrophages Negatively Affects M1 Polarization and Inflammatory Responses

**DOI:** 10.3389/fimmu.2022.782475

**Published:** 2022-03-29

**Authors:** So-Yeon Choi, Yu Ri Ahn, Eun-Bi Lee, Mi Jin Yu, Jong Ran Lee

**Affiliations:** ^1^Department of Bioinspired Science, Graduate School, Ewha Womans University, Seoul, South Korea; ^2^Department of Life Science, College of Natural Sciences, Ewha Womans University, Seoul, South Korea

**Keywords:** p190RhoGEF, macrophage, lipopolysaccharide, actin cytoskeleton, M1 polarization, RhoA

## Abstract

A RhoA-specific guanine nucleotide exchange factor, p190RhoGEF, was first cloned and identified in neuronal cells. In immune cells, we first reported the role of p190RhoGEF in B cells: expression of p190RhoGEF increased after CD40 stimulation and was required for CD40-mediated B cell activation and differentiation. We also showed that over-expression of p190RhoGEF negatively affected dendritic cell function in response to bacterial lipopolysaccharide (LPS). In this study, we examined the role of p190RhoGEF in macrophages using p190RhoGEF over-expressing transgenic (TG) mice. We found macrophages from TG mice to be more round than those from control mice, with enriched polymerized actin at the edge attached to the glass. TG macrophages also responded less to LPS: production of reactive oxygen species, phagocytosis, chemokine-dependent migration, and pro-inflammatory cytokine secretion were all reduced compared with the responses of macrophages from littermate (LTM) control mice. Furthermore, the classical M1 subset population was observed less in the peritoneal macrophages of TG mice than the LTM control mice during LPS-elicited peritoneal inflammation. When the activity of RhoA was inhibited in TG macrophages, their morphology and LPS responses became similar to those of the LTM macrophages. These results suggest that over-expression of p190RhoGEF in macrophages could reduce M1 polarization and inflammatory responses by regulating the actin cytoskeleton.

## Introduction

A RhoA-specific guanine nucleotide exchange factor, p190RhoGEF, was shown to be expressed ubiquitously (1). It was first cloned in neuronal cells and was demonstrated to function in controlling neuronal morphology (1–3). Over-expression of this protein mimicked activated RhoA in stimulating cytoskeletal contraction, preventing neurite out-growth (1, 2). Previously, we were the first to report the role of this protein in immune cells. Our studies in B cells demonstrated that the expression of p190RhoGEF increased dramatically following CD40 stimulation, producing changes in cellular morphology, transcriptional regulation, and B cell differentiation during CD40-mediated B cell activation (4–6). We also showed that over-expression of p190RhoGEF or constitutive activation of RhoA mimicked the effects of CD40 stimulation in B cells (4).

To further examine the physiological role of p190RhoGEF, we developed transgenic (TG) mice in which transgenes are specifically expressed in antigen presenting cells (APCs) with class II major histocompatibility complex (MHC) (7). Our studies in the TG mice showed that over-expression of p190RhoGEF in CD11c^+^ conventional dendritic cells (DCs) negatively regulated cellular responses to bacterial lipopolysaccharide (LPS) (8). In this study, we examine the role of p190RhoGEF in macrophages, which also express class II MHC molecules. Macrophages are recruited to inflamed areas and play key roles in inflammation by acquiring distinct functional phenotypes, either classically activated, pro-inflammatory macrophages (M1) or alternatively activated, anti-inflammatory macrophages (M2) (9–12).

RhoA activation is important for the regulation of the actin cytoskeleton, which is crucial to multiple cellular processes essential for an optimal immune response (13–17). Regulating the actin cytoskeleton through the RhoA pathway has been shown to change macrophage shapes and functions (18, 19). Previous reports also showed that phenotypically and functionally distinct subtypes of macrophages have characteristic shapes: M1 subtypes are roundish, and M2 subtypes are elongated (18, 20, 21).

In the present study, we compared peritoneal macrophages (PMs) and bone marrow-derived macrophages (BMDMs) isolated from littermate (LTM) and p190RhoGEF TG mice. We examined whether the over-expression of p190RhoGEF changes the shape of macrophages, consequently macrophage subset polarization in LTM and p190RhoGEF TG mice. We also examined whether the changes in cellular morphology that are regulated by the over-expression of p190RhoGEF correlate with the function of macrophages. We found more cells from the TG mice to be roundish in shape, with polymerized actin concentrated at the edge attached to the slide. The TG macrophages also showed reduced responses to LPS. These features of the TG macrophages were reduced when the activity of RhoA was inhibited: the shape and LPS responses became similar to those of the LTM macrophages. These results suggest that over-expression of p190RhoGEF could suppress the pro-inflammatory functions of macrophages by regulating the actin cytoskeleton.

## Materials and Methods

### Antibodies (Abs) and Reagents

The following monoclonal Abs (mAbs) were purchased from eBioscience, Inc. (Santa Clara, CA): peridinin chlorophyll protein (PerCP)-conjugated rat anti-mouse F4/80 (BM8), r-phycoerythrin (PE)-conjugated rat anti-mouse CD11b (M1/70), biotin-conjugated rat anti-mouse CD86 (B7-2) (GL1), and fluorescein isothiocyanate (FITC)-conjugated rat anti-mouse CD11c (N418). FITC-conjugated rat anti-mouse CD40 was purchased from BD Biosciences (San Diego, CA). Allophycocyanin (APC)-conjugated rat anti-mouse CD206 (MMR) and APC- or Brilliant Violet (BV) 421-conjugated rat anti-mouse CD163 were from BioLegend (San Diego, CA). Isotype control IgGs were purchased from Jackson ImmunoResearch Laboratories, Inc. (West Grove, PA). Ready-SET-Go cytokine ELISA sets for mouse IL-1β, TNF-α, and IL-6 were obtained from eBioscience, Inc. A polyclonal anti-mouse p190RhoGEF Ab (LF-r-gef) was described previously (5). A mouse mAbs for β-actin (C4), RhoA (67B9), glutathione *S*-transferase (GST, B-14) were purchased from Santa Cruz Biotechnology, Inc. (Santa Cruz, CA). Horseradish peroxidase (HRP)-conjugated anti-rabbit IgG and anti-mouse IgG (Bio-Rad, Hercules, CA) were used as secondary Abs for immunoblotting (IB). Enhanced chemiluminescence (ECL) reagents from Amersham Pharmacia Biotech Co. (Arlington Heights, IL) were used. TRIzol reagent was purchased from Life Technologies (Grand Island, NY), and Oligo(dT)_12-18_ primer and Moloney murine leukemia virus reverse transcriptase were purchased from Promega (Madison, WI). DAPI (4′,6-diamidino-2-phenylindol dihydrochloride) was purchased from Roche Diagnostics GmbH (Mannheim, Germany). FITC- or rhodamine-phalloidin, LPS (*Escherichia coli*, serotype 055:B5), lucigenin, glutathione-agarose, and FITC-zymosan were purchased from Sigma-Aldrich Co. (St. Louis, MO). N-formylmethionine-leucyl-phenylalanine (fMLP) and rhosin from Calbiochem (La Jolla, CA) and 2’, 7’-dichloro-dihydrofluorescein diacetate (DCFDA) from Molecular Probes (Eugene, OR) were used. Recombinant murine granulocyte-macrophage colony-stimulating factor (GM-CSF, 315-03) was obtained from PeproTech (Cranbury, NJ).

### Mice and LPS Injection

The generation of the p190RhoGEF TG mice was described previously (8). Mice were bred and maintained under specific pathogen–free conditions at the animal facility of the Ewha Laboratory Animal Genomic Center in accordance with institutional guidelines. Eight- to fourteen-week-old mice were used for all experiments. Mice were injected intraperitoneally with LPS (6 mg/kg) for 4 hours. The control animals were injected with the same volume of phosphate-buffered saline (PBS). All mouse protocols were approved by the Ewha Institutional Animal Care and Use Committee.

### Isolation of PMs

The peritoneal cavity was lavaged by injecting 1 ml of sterile PBS. Cells were harvested and resuspended in RPMI culture medium. Macrophages were allowed to adhere onto the culture dish overnight at 37°C in a 5% CO_2_ incubator, and non-adherent cells were removed by washing twice with PBS. Macrophages collected using a scraper were washed twice with PBS before experiments.

### BMDM Preparation

Bone marrow cells were flushed from the femurs and tibiae of mice, as described previously (22). Those cells were cultured in RPMI 1640 medium containing 10% FBS and 10 ng/ml murine GM-CSF for 5–7 days. Cell purity was determined by FACS analysis for CD11b (> 96%).

### RNA Extraction and RT-PCR

Total RNA was extracted from purified macrophages using TRIzol reagent. Complementary DNA was prepared from an RNA template with oligo(dT) primers and was subjected to PCR. A fragment of either p190RhoGEF or glyceraldehyde-3-phosphate dehydrogenase (GAPDH) was amplified, and the PCR products were separated on a 1.2% agarose gel. The primer sequences used were as follows. p190RhoGEF: 5’-GCCTCTAGATCTTCTCTGTGGATCGAC-3’, 5’-TCCAGCAGCCATCTAAGCAGG-3’; GAPDH: 5´-ATCACCATCTTCCAGGAGCGA-3´, 5´-ATGACCTTGCCCACAGCCTT-3´.

### Quantitative Real-Time PCR

Quantitative real-time PCR (Q-PCR) was performed as described previously (6). An aliquot of cDNA template was amplified in a final volume of 10 μl of reaction mixture containing SYBR Green PCR Master Mix and each gene-specific primer using a StepOne Real-Time PCR System (Applied Biosystems, San Diego, CA). The fluorescence signals were quantified by the comparative cycle threshold method and normalized with GAPDH mRNA as an internal control gene. The primer sequences used were as follows. IL-6: 5′-GACTGATGCTGGTGACAACC-3′, 5′-AGACAGGTCTGTTGGGAGTG-3′; IL-1β: 5′-GACCTGTTTGAAGTTGACG-3′, 5′-GCGAGATTTGAAGCTGGATG-3′; TNF-α: 5′- GATGAGAAGTTCCCAAATGGC-3′, 5′-CAGGCTTGTCACTCGAATTTTG-3′; GAPDH: 5′-TCACGGCAAATTCAACGGCACA-3′, 5′-ATGGGCTTCCCGTTGATG ACAA-3′.

### IB Analysis

IB was performed as described previously (5). Whole cell lysates of BMDMs were prepared in 1% Nonidet P-40 lysis buffer containing protease and phosphatase inhibitors; 50 μg of the lysates were then subjected to 6% SDS-PAGE. IB analysis was performed with an anti-mouse p190RhoGEF polyclonal Ab (LF-r-gef) or an anti-mouse β-actin Ab, followed by an HRP-conjugated secondary Ab. Chemiluminescent detection was conducted with ECL reagents.

### Morphology and Actin Microscopy

Macrophages (2 x 10^5^) were incubated in a petri dish overnight. The morphology of the macrophages attached to the dish was observed under a microscope with a 20X or 40X objective lens (Axiovert 200, Carl Zeiss, Inc.) at Ewha Fluorescence Core Imaging Center. Morphological differences were enumerated in 3 different fields in each sample, and the percentage of cells well spread (or the cellular area) was calculated. The area of each individual cell was determined using Image J analysis software. Macrophages (1 x 10^5^) attached on a cover glass were fixed with 4% paraformaldehyde for 10 minutes and permeabilized with 0.1% Triton X-100 for 10 minutes at room temperature. After being blocked with 1% BSA for 1 hour, the samples were incubated with FITC- or rhodamine-conjugated phalloidin for 1 hour and then with DAPI for 5 minutes at room temperature. After being mounted, the samples were analyzed using a Zeiss Axiovert 200 inverted microscope that was equipped with an X40 Achroplan LD or X20 Plan-Neofluar objective, a Zeiss AxioCam HRc, and an Axio Vision Rel. 4.8 software (Carl Zeiss, Oberkochen, Germany).

### Flow Cytometry

Single-cell suspensions of isolated macrophages (2 x 10^5^/ml) were washed, blocked with the appropriate sera, and stained for specific cell surface markers as described previously (5). To stain actin, cells were fixed and permeabilized. F-actin was stained using FITC- or rhodamine-phalloidin. Three- or four-color flow cytometric analyses were then conducted for these cells. The data were acquired on a FACSCalibur or an LSR Fortessa cell analyser using CELLQuest software (BD Biosciences, San Jose, CA) at Ewha Fluorescence Core Imaging Center. Fluorescence signals were analyzed as dot plots of the fluorescence intensity. The percentage of the cell population in the gated area is represented graphically in the figures.

### ROS Measurements

Intracellular ROS were measured using a fluorescent probe, DCFDA (23). Macrophages (2.5 x 10^5^) were incubated in serum-free medium for 1 hour at 37°C and then stimulated with LPS (100 ng/ml) for 30 minutes. DCFDA (5 μM) was added and incubated for 15 minutes before the fluorescent cells were read using a FACS. ROS production by macrophages (1 x10^6^) that followed LPS (1 μg/ml) treatment was also measured using a Nikon Eclipse Ts2R-FL inverted microscope with a 40X objective lens at Ewha Fluorescence Core Imaging Center. Fluorescence intensities were measured under excitation settings at 488 nm and emission at 515–540 nm. Fluorescence cell intensities that were collected with identical parameters, such as contrast and brightness, were compared between the control and LPS stimulation conditions. To measure ROS release in the media, macrophages were cultured in black 96-well plates (1.5 x10^5^/well) overnight and stimulated with either PBS or LPS (100 ng/ml). After adding lucigenin (200 μM), luminescence was measured for 60 minutes at 2 minute-intervals by a SpectraMax 190 microplate reader (Molecular Devices, Sunnyvale, CA).

### Phagocytic Activity Assay

Macrophages (2.5 x 10^5^/ml) were starved in serum-free RPMI medium for 1 hour, and FITC-zymosan particles (20 μg/ml) were added. After being incubated at 37°C for 30 minutes, the cells were washed three times in PBS, and the FITC intake was analyzed using a FACS.

### *In Vitro* Migration Assay

An *in vitro* chemotaxis assay was performed using Costar Transwell inserts (5 μm pores) in 24-well plates as previously described (8). Macrophages (2.5 x 10^5^) suspended in serum-free RPMI medium were put in the upper Transwell insert in a volume of 100 μl. The lower well contained 600 μl of serum-free RPMI medium containing a chemoattractant, fMLP (1 mM). The plates were incubated at 37°C for 2 hours, and the cells that migrated to the lower chamber were harvested and counted.

### ELISA

Ready-SET-Go cytokine ELISA sets were used according to the manufacturer’s instructions. Macrophages (2.5 x 10^4^) were stimulated with 200 μl of medium in the presence or absence of LPS (100 ng/ml or 1 μg/ml) in a 96-well U-bottomed culture plate for 24 hours or 1 hour. For IL-1β ELISA, cells were stimulated with ATP (2 mM) for 1 hour after 3-hour LPS (100 ng/ml) treatment. The culture supernatants collected were added to each well of 96-well flat-bottomed ELISA plates that were prepared for the assay. At the end of the assay, the plates were read at 450 nm using a microplate reader (Molecular Devices, Spectra MAX 190).

### RhoA Activation Assay

GST fusion protein containing the Rho-binding domain of Rhotekin (RBD-GST, the plasmid from Dr. G. Schmidt, Albert-Ludwigs-Universität Freiburg, Germany), which specifically recognizes the GTP-bound form of Rho proteins (24), was prepared as described elsewhere (25), and bound to glutathione-coated agarose beads. Lysates (400 μg) of PMs from LTM and TG mice were incubated for 1 hour at 4°C with 10 μg of RBD-GST fusion proteins immobilized on 10 μl of glutathione-coated beads. Proteins bound to the fusion proteins were separated by 8% SDS-PAGE and transferred to nitrocellulose membrane for IB with anti-RhoA, anti-GST, and anti-β-actin Abs.

### Statistical Analysis

Comparisons of multiple conditions were performed using one-way ANOVA with *post hoc* tests. Statistics were determined using Prism software (GraphPad Software, Inc., San Diego, CA). Values of *p* < 0.05 were considered significant. Asterisks indicate significant differences between different groups (*p < 0.05; **p < 0.01; ***p < 0.001).

## Results

### Macrophages From p190RhoGEF TG Mice Show Roundish Shapes With Polymerized Actin Enrichment at The Edge

**Figure 1:**
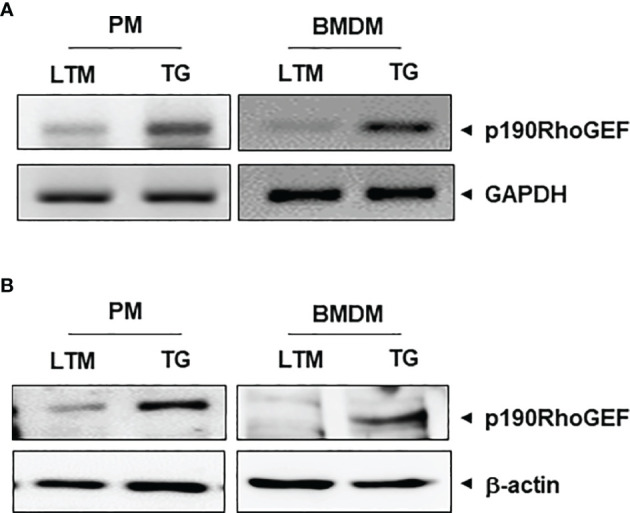
p190RhoGEF transgene expression in TG macrophages. **(A)** mRNA transcripts of p190RhoGEF in PMs and BMDMs from LTM and TG mice. GAPDH was used as a control. **(B)** Expression analysis of p190RhoGEF protein in PMs and BMDMs from LTM and TG mice. Actin was used as a control. All data are representative of three separate experiments.

Using p190RhoGEF TG mice that highly express the transgene in cells with class II MHC molecules, we previously reported that p190RhoGEF transgene is expressed in DCs that express class II MHC molecules (8). In this work, we tested whether p190RhoGEF transgene is also expressed in the macrophages of TG mice because macrophages also express class II MHC molecules. PMs were isolated from peritoneal cavity lavage of LTM control and p190RhoGEF over-expressing TG mice by removing non-adherent cells. BMDMs were also prepared from LTM control and p190RhoGEF TG mice. The transcriptional levels of p190RhoGEF were compared by RT-PCR (**Figure 1A**), and the protein expression was analyzed by Western blotting on macrophage cellular extracts using an anti-p190RhoGEF Ab (**Figure 1B**). As shown in the splenic DCs (8), endogenous expression of p190RhoGEF was also seen in macrophages isolated from the LTM control mice, and the over-expression of the p190RhoGEF transgene was confirmed in the macrophages isolated from the TG mice.

**Figure 2:**
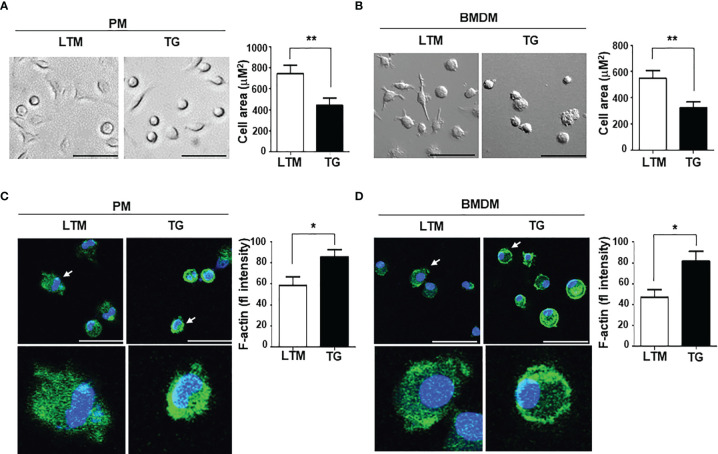
Morphological characteristics associated with p190RhoGEF transgene expression in TG macrophages. **(A, B)** Morphological comparison of PMs and BMDMs from LTM and TG mice. Scale bars = 50 μm. Cellular area was measured in each experiment and the data were compared as the mean ± SEM. Statistical significance was analyzed by unpaired t tests. Significant comparisons are noted as **p < 0.01. **(C, D)** Actin and nuclei staining with FITC-phalloidin (green) and DAPI (blue), respectively, in PMs and BMDMs from LTM and TG mice. All are merged images of actin staining and DAPI. Scale bars = 50 μm. An enlarged image of single cell (indicated by an arrow) is also shown. Green fluorescence (fl) intensities of cells in each experiment were analyzed and the data were compared as the mean ± SEM. Statistical significance was analyzed by unpaired t tests. Significant comparisons are noted as *p < 0.05. All data are representative of five separate experiments.

We examined the macrophage morphology and found that macrophages from the LTM and p190RhoGEF TG mice showed differences in shape. The LTM macrophages were spread well enough to show elongation, but the TG macrophages were roundish (**Figures 2A, B**). Polymerized actin was enriched at the edges of the attached TG macrophages (**Figures 2C, D**). These results could suggest that the p190RhoGEF over-expressed in TG macrophages activated changes in the RhoA-mediated actin cytoskeleton, which controls macrophage shape. We next examined whether these actin cytoskeletal changes also affected the macrophage subset population in the peritoneum of TG mice.

### Reduced M1 Polarization Is Seen in p190RhoGEF TG Mice During Peritoneal Inflammation

**Figure 3:**
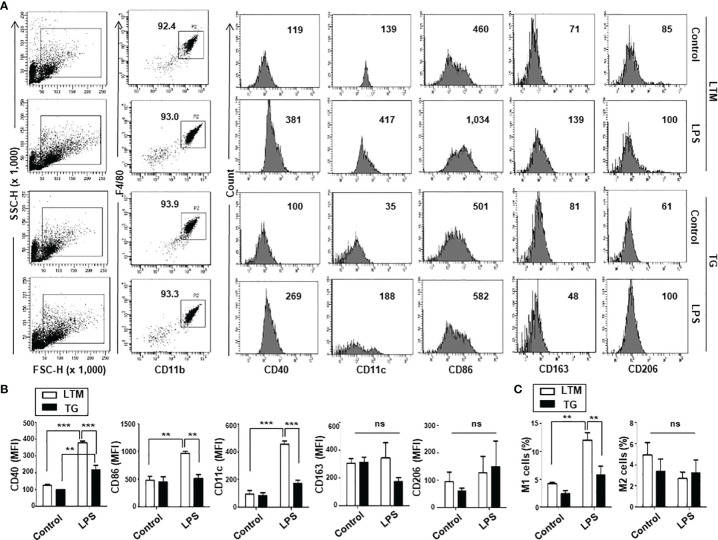
Macrophage subset populations in LTM and TG mice. Analyses of subset populations of PMs from LTM and TG mice in normal conditions and during peritoneal inflammation caused by LPS. PMs were labeled with FITC- or APC-conjugated anti-CD11c, FITC-conjugated anti-CD40 or anti-CD86, BV421- or APC-conjugated CD163, APC-conjugated CD206 mAbs in color combinations, along with mAbs for PE-conjugated CD11b and PerCP-conjugated F4/80. Four-color flow cytometric analyses were then conducted. **(A)** Representative results are presented as dot plots, showing F4/80 *vs.* CD11b staining, and the percentages of the total cells that they represent are indicated. The expression of M1 markers (CD40, CD11c, CD86) and M2 markers (CD163, CD206) of CD11b^+^F4/80^+^ cells is shown as the mean fluorescence intensity (MFI) of each marker. **(B)** The MFI of each marker is compared and the data from three independent experiments are presented as the mean ± SEM. Statistical significance was analyzed by one-way ANOVA with Tukey’s multiple comparison tests. Significant comparisons are noted as **p < 0.01, ***p < 0.001, ns, not significant. **(C)** Changes in the M1/M2 subset population during peritoneal inflammation caused by LPS were compared as the percentage of CD11b^+^F4/80^+^ cells expressing CD11c (M1) and the percentage of CD11b^+^F4/80^+^ cells expressing CD163 (M2). Data shown are the mean ± SEM of three separate analyses. Significance was determined by one-way ANOVA with Tukey’s multiple comparison tests (**p < 0.01, ns, not significant).

Flow cytometry analyses of PMs from LTM and p190RhoGEF TG mice showed that the total population of cells expressing F4/80 and CD11b (CD11b^+^F4/80^+^) was similar (**Figure 3A**), and subset populations (M1: CD11b^+^F4/80^+^CD11C^+^, M2: CD11b^+^F4/80^+^CD163^+^) were smaller in the PMs from TG mice, although the difference was not statistically significant due to individual variation among mice (**Figure 3C**). However, the ratio of subset populations of PMs was maintained similarly in LTM and TG mice (data not shown). To analyze the macrophage response during peritoneal inflammation elicited by LPS, changes in M1/M2 subset polarization were compared. Flow cytometry analyses showed that the M1 population (CD11b^+^F4/80^+^CD11C^+^) was significantly increased in the LPS-stimulated PMs of the LTM control mice, but not in those of the p190RhoGEF TG mice (**Figures 3C**). As a result, the LPS-stimulated PMs of the TG mice had a significantly smaller population of M1 subset PMs than the LTM control mice (**Figures 3C**). The M2 population (CD11b^+^F4/80^+^CD163^+^) was not significantly changed in the LPS-stimulated PMs of the LTM and the TG mice (**Figure 3C**). The expression of M1 markers was also significantly increased in the LPS-stimulated PMs of the control mice, but not in those of the TG mice (**Figures 3A, B**). Significant changes in the expression of M2 markers were not shown (**Figures 3A, B**). Those results suggest that over-expression of p190RhoGEF in macrophages might lead to a reduction in macrophage polarization toward the classical M1 subset during inflammation.

### TG Macrophages Show Reduced Responses to LPS

Previous studies indicated that M1 macrophages are roundish (12, 20, 21) and that macrophage shapes and functions depend on an actin cytoskeleton that is regulated by RhoGEF/RhoA activation pathways (18, 19). We therefore compared the characteristics of the M1 subset in macrophages from LTM and TG mice. Macrophages are polarized to the M1 phenotype in the presence of Th1 cytokines such as IFN-γ or bacterial products such as LPS (26, 27). M1 macrophages produce high levels of inflammatory cytokines such as TNF-α, IL-6, IL-1β, IL-12, and IL-23, as well as increased levels of ROS (10–13).

**Figure 4:**
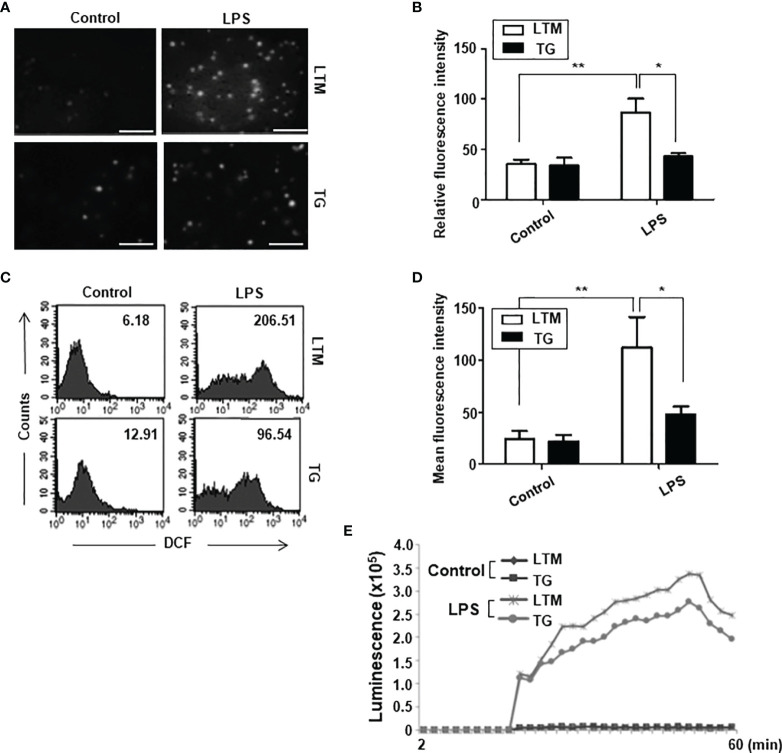
Analyses of ROS production in the PMs of LTM and p190RhoGEF TG mice. **(A)** Images show LPS-mediated ROS production in PMs from LTM and TG mice. PMs (1 x10^6^) that were loaded with 5 μM DCFDA for 15 minutes were stimulated with LPS (1 μg/ml). Representative microscopic fields of DCF fluorescence from the cells after a 30-minute stimulation with either control (PBS) or LPS are shown. Scale bars = 50 μm. **(B)** The DCF fluorescence intensity of PMs from LTM and TG mice after a 30-minute stimulation with LPS is shown. The data are the averaged intensities from three microscopic fields (10 cells in each field). The data are presented as the mean ± SEM. Statistical significance was analyzed by one-way ANOVA with Tukey’s multiple comparison tests. Significant comparisons are noted as *p < 0.05, **p < 0.01. The experiments were repeated three times. **(C)** LTM and TG PMs (2.5 x10^5^) were loaded with 5 μM DCFDA for 15 minutes and then stimulated with LPS (100 ng/ml) for 30 minutes. The DCF fluorescent cells were measured using flow cytometry. The numbers indicate the mean fluorescence intensity (MFI). The data shown are representative of at least five separate experiments. **(D)** The MFI of PMs from LTM and TG mice after a 30-minute stimulation with LPS is compared and the data are presented as the mean ± SEM. Statistical significance was analyzed by one-way ANOVA with Tukey’s multiple comparison tests (*p < 0.05, **p < 0.01). **(E)** LTM and TG PMs cultured in a black 96-well plate (1.5 x10^5^/well) were stimulated with LPS (100 ng/ml). Luminescence was detected 2 minutes after adding lucigenin (200 μM) for 60 minutes at 2-minute intervals. Experiments were repeated twice with similar results.

We first examined whether the expression of the p190RhoGEF transgene in macrophages affected their ability to produce ROS. To test that, PMs prepared from LTM control and p190RhoGEF TG mice were treated with LPS. Changes in the production of intracellular ROS were examined using DCF dye, which fluoresces in response to ROS. Representative fluorescence images of cells after a 30-minute stimulation are shown (**Figure 4A**); the fluorescence intensity is compared quantitatively (**Figure 4B**). The fluorescence in cells was also determined by flow cytometry (**Figure 4C**); the mean fluorescence intensity (MFI) is compared quantitatively (**Figure 4D**). These results demonstrate that ROS production was dramatically reduced in PMs from TG mice compared with that in cells from LTM control mice when cells were activated for 30 minutes with LPS. Similar results were also obtained by chemiluminescence detection using lucigenin when PMs from LTM control and p190RhoGEF TG mice were incubated in medium containing LPS. The luminescence caused by ROS release into the medium appeared 20 minutes after the stimulation, increased continuously for another 30 minutes, and then started to fade (**Figure 4E**). Less luminescence was observed from the PMs of the TG mice than in those from the LTM control mice (**Figure 4E**).

**Figure 5:**
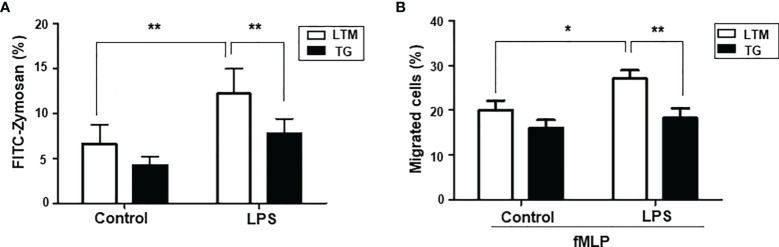
Phagocytic and chemotactic ability of PMs from LTM and p190RhoGEF TG mice. PMs from LTM and TG mice were cultured in media with or without LPS (100 ng/ml) overnight. These PMs were used for the assay. **(A)** PMs (2.5 x 10^5^) were incubated with FITC-conjugated zymosan (20 μg/ml) at 37**°**C for 30 minutes. The PM population made fluorescent by zymosan internalization was detected using flow cytometry. The data are the mean ± SEM of five independent experiments. Statistical significance was analyzed by one-way ANOVA with Tukey’s multiple comparison tests. Significant comparisons are noted as **p < 0.01. **(B)** PMs (2.5 x 10^5^) were added to the upper wells of a Transwell plate that contained the chemoattractant fMLP (1 mM) in the lower chambers. The cells that migrated to the lower chamber were counted after the plate was incubated at 37**°**C for 2 hours. The data are presented as the percentage of the total cells that migrated. The data shown are the mean ± SEM of three independent experiments with duplicate sample settings. Statistical significance was analyzed by one-way ANOVA with Tukey’s multiple comparison tests. Significant comparisons are noted as *p < 0.05, **p < 0.01.

We next examined whether p190RhoGEF transgene expression altered the abilities of macrophages to engulf particles. PMs from the LTM control and p190RhoGEF TG mice were incubated with FITC-conjugated zymosan at 37°C for 30 minutes, and then the phagocytic capacity of the macrophages was compared. The macrophage population made fluorescent by zymosan internalization increased after LPS treatment, and that increase was significantly smaller in PMs from the p190RhoGEF TG mice than in the PMs from the LTM control mice (**Figure 5A**). To compare the chemotactic ability of PMs from LTM and TG mice, we analyzed the cells that migrated toward fMLP in the lower chamber of a Transwell plate. Macrophage migration increased significantly after LPS stimulation in the LTM macrophages, but not in the TG macrophages (**Figure 5B**).

**Figure 6:**
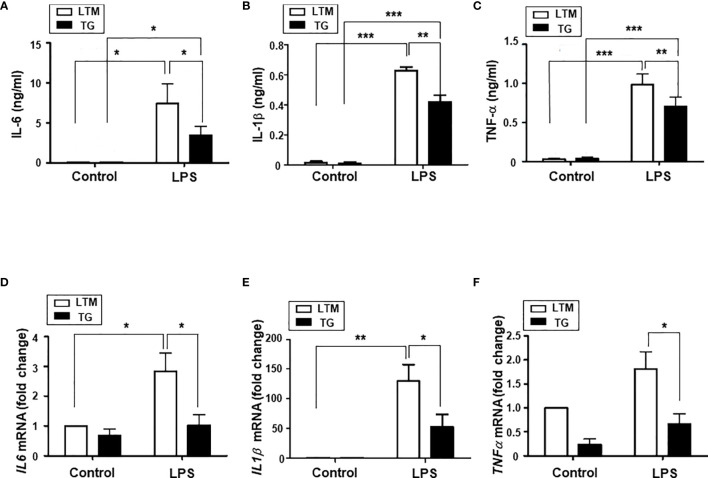
Analyses of inflammatory cytokine production in the PMs of LTM and p190RhoGEF TG mice. PMs from LTM control and TG mice were cultured in the medium alone (control) or with LPS (100 ng/ml) overnight. For IL-1β ELISA, cells were stimulated with ATP (2 mM) for 1 hour after 3-hour LPS (100 ng/ml) treatment. **(A–C)** The levels of IL-6, IL-1β, and TNF-α in the supernatants were compared by ELISA. The data are the mean ± SEM of three independent experiments with triplet sample settings. Statistical significance was analyzed by one-way ANOVA with Tukey’s multiple comparison tests. Significant comparisons are noted as *p < 0.05, **p < 0.01, ***p < 0.001. **(D–F)** Messenger RNA transcripts of IL-6, IL-1β, and TNF-α were compared by quantitative real-time PCR analysis, as described in Materials and Methods. The relative level of expression is shown compared with the expression level of LTM control PM cells. Data are the mean ± SEM of four separate experiments. Significance was determined by one-way ANOVA with Tukey’s multiple comparison tests. Significant comparisons are noted as *p < 0.05, **p < 0.01.

We further studied the production of inflammatory cytokines, IL-6, IL-1β; and TNF-α; in PMs from LTM control and p190RhoGEF TG mice. The secretion of those pro-inflammatory cytokines increased significantly after stimulation with LPS (**Figures 6A–C**). However, the level of secretion in response to LPS was reduced significantly in the TG macrophages compared with the LTM control macrophages (**Figures 6A–C**). LPS-induced cytokine gene transcription was also reduced significantly in the PMs of TG mice (**Figures 6D–F**). These results indicate that the reduction of cytokine secretion in TG macrophages after LPS treatment was caused by a reduced level of cytokine gene transcription.

### Inhibiting RhoA in TG Macrophages Returns Their Shape and Function Back to Those of Control Macrophages

**Figure 7:**
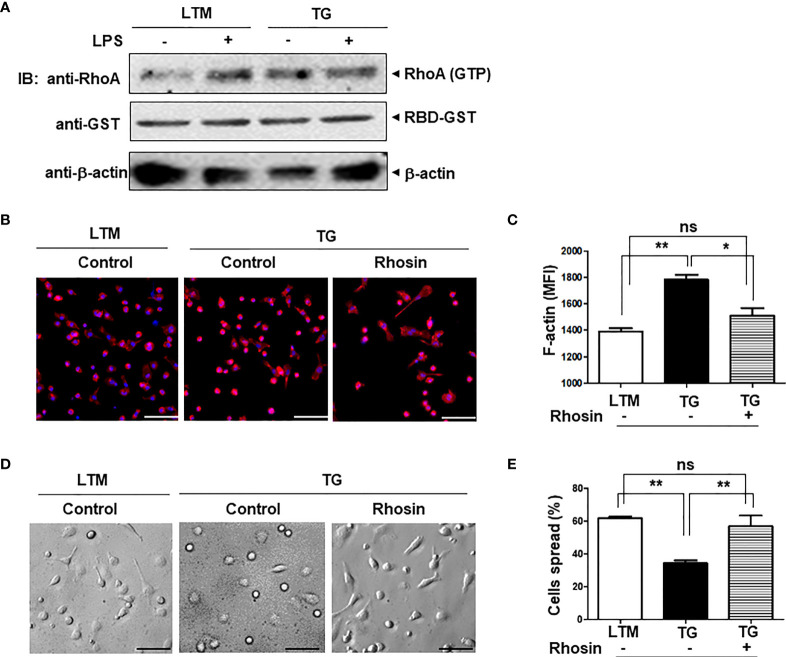
Morphological changes in p190RhoGEF TG macrophages caused by the inhibition of RhoA. **(A)** PMs from LTM control and p190RhoGEF TG mice were either left unstimulated, or stimulated with LPS (1 μg/ml) at 37**°**C for 15 minutes. Cell lysates (400 μg) were incubated with rhotekin RBD-GST fusion protein beads (10 μg) at 4**°**C for 1 hour. Association of GTP-bound RhoA was determined with anti-RhoA Ab. Amounts of beads and lysates used in each sample were shown by IB with anti-GST and anti-β-actin Abs, respectively. Experiments were repeated twice with similar results. **(B)** LTM and TG PMs were attached to cover glass overnight. TG PMs were treated with an inhibitor of RhoA, rhosin (1 μM) for 30 minutes. Actin and nuclei were stained with rhodamine-phalloidin (red) and DAPI (blue), respectively. Merged images of actin and DAPI staining are shown. The cells were analyzed under a fluorescent microscope. Scale bars = 50 μm. **(C)** Fluorescence signals of F-actin stained with phalloidin were analyzed by flow cytometry, and the MFI was compared. **(D, E)** PMs from LTM control and p190RhoGEF TG mice were attached to cover glass overnight. TG PMs were treated with an inhibitor of RhoA, rhosin (1 μM) for 30 minutes. Morphological comparisons under a microscope are shown. Scale bars = 50 μm **(D)**. Morphological differences were enumerated in 3 different fields in each sample, and the percentage of cells well spread was compared **(E)**. All data are representative of five separate experiments. The data are the mean ± SEM. Statistical significance was analyzed by one-way ANOVA with Tukey’s multiple comparison tests. Significant comparisons are noted as *p < 0.05, **p < 0.01, ns, not significant.

**Figure 8:**
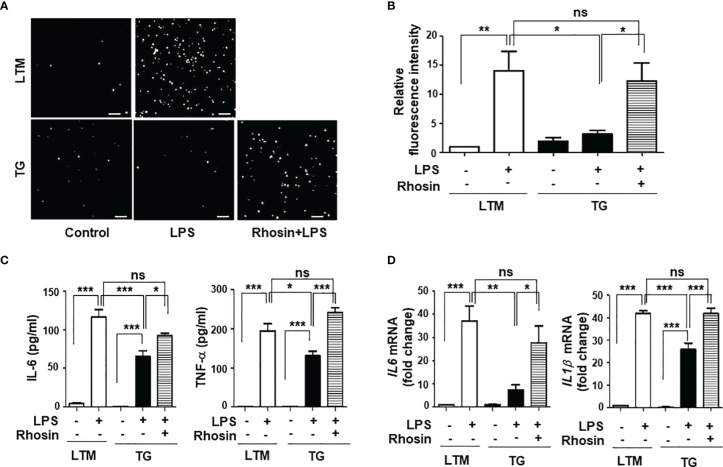
Functional changes in p190RhoGEF TG macrophages caused by the inhibition of RhoA. TG PMs were pre-treated with an inhibitor of RhoA, rhosin (1 μM) for 1 hour. **(A, B)** LPS-mediated ROS production was compared as described in **Figures 4A, B**. Representative images (scale bars = 50 μm) **(A)** and the DCF fluorescence intensity **(B)** of PMs from LTM and TG mice after a 30-minute stimulation with LPS (1 μg/ml) are shown. The data from three different experiments are presented as the mean ± SEM. Statistical significance was analyzed by one-way ANOVA with Tukey’s multiple comparison tests. Significant comparisons are noted as *p < 0.05, **p < 0.01, ns, not significant. **(C, D)** PMs from LTM control and TG mice were incubated in medium alone (control) or with LPS (1 μg/ml) for 1 hour. The levels of IL-6 and TNF-α in the supernatants were compared by ELISA **(C)**. The data are the mean ± SEM of three independent experiments with triplet sample settings in each experiment. Statistical significance was analyzed by one-way ANOVA with Tukey’s multiple comparison tests. Significant comparisons are noted as *p < 0.05, ***p < 0.001, ns, not significant. Messenger RNA transcripts of IL-6 and IL-1β were compared by quantitative real-time PCR analysis **(D)**. The relative level of expression is compared with the expression level in LTM control PM cells. Data are the mean ± SEM of three separate experiments. Statistical significance was analyzed by one-way ANOVA with Tukey’s multiple comparison tests. Significant comparisons are noted as *p < 0.05, **p < 0.01, ***p < 0.001, ns, not significant.

p190RhoGEF was identified as a specific GEF for RhoA activation, and RhoA is important in the regulation of the actin cytoskeleton (1, 14, 15, 28). We examined the level of RhoA activation in LTM and TG macrophages and found that endogenous level of RhoA activation in TG macrophages was similar to the RhoA activation stimulated by LPS in LTM macrophages (**Figure 7A**). Compared with LTM macrophages, TG macrophages showed little response to LPS (**Figure 7A**). To determine whether the characteristics of shape, subset polarization, and LPS response shown in the TG macrophages are caused by the over-expression of p190RhoGEF, we tested the effect of an inhibitor for RhoA, rhosin, which directly targets the Rho-GEF binding domain, preventing Rho from interacting with its GEFs (29, 30). After the rhosin treatment, the level of polymerized actin in the TG macrophages was reduced to that of the LTM macrophages (**Figures 7B, C**). Consequently, the TG macrophages were spread better, as in the LTM control macrophages, by the rhosin treatment (**Figures 7D, E**). Furthermore, LPS-induced ROS production and inflammatory cytokine gene transcription in the TG macrophages also recovered after rhosin treatment (**Figures 8A–D**). The effect of rhosin alone was similar to the control (data not shown). These results suggest that the over-expression of p190RhoGEF might regulate TG macrophages structurally and functionally.

## Discussion

Macrophages are major components of the innate immune system, which triggers inflammatory responses, and they play essential roles in establishing pro- or anti-inflammatory environments (11, 12, 31). Multiple macrophage phenotypes have been demonstrated, and their differential functions have various effects on surrounding cells that regulate the immune responses (11–13, 26, 27). Various intrinsic microenvironments, such as differentiation signaling pathways and cytokine inputs, work together to drive the functional diversity of macrophages (13, 26, 27).

We previously identified p190RhoGEF as a protein whose expression is dramatically increased in B cells following CD40 stimulation (4). We showed that p190RhoGEF over-expression or constitutive activation of RhoA mimicked the effects of CD40 stimulation in B cells (4). Our studies in B cells also showed that the changes in cellular morphology that are regulated by p190RhoGEF during CD40-mediated B cell activation correlate with the expression of surface molecules and transcriptional regulators required for B cell maturation and differentiation (5, 6). We created TG mice in which the expression of p190RhoGEF is driven by an invariant chain (Ii) promoter to further examine the role of p190RhoGEF *in vivo*. Thus, transgenes are specifically expressed in APCs with class II MHC, including B cells, macrophages, and DCs. Using these TG mice, we demonstrated that the over-expression of p190RhoGEF negatively regulates DC function in response to LPS (8).

In this study, we examined the effects of p190RhoGEF over-expression in the macrophages of TG mice. As seen in the DCs, endogenous expression of p190RhoGEF was found in PMs and BMDMs at the RNA and protein levels. Interestingly, macrophages with p190RhoGEF transgene over-expression were roundish in shape, with polymerized actin enriched at the edges of macrophages attached to the slide. Previous reports showed that phenotypically and functionally distinct subtypes of macrophages have characteristic shapes: M1 subtypes are roundish, and M2 subtypes are elongated (18, 20, 21). We inquired whether macrophage subtypes differed between LTM and TG macrophages. Flow cytometry analyses of PMs from LTM and p190RhoGEF TG mice showed a similar ratio of M1/M2 populations, although variations in subset populations appeared among individual mice.

To analyze further the responses of macrophages *in vivo*, peritoneal inflammation was induced by LPS injection. That inflammatory condition induced M1 subset polarization in the LTM macrophages, but that reaction was significantly reduced in the TG macrophages. We also examined whether macrophages from LTM control and TG mice respond similarly to LPS treatment. Interestingly, LPS-derived functions (ROS production, phagocytosis, chemotaxis, and inflammatory cytokine secretion) were all reduced in the p190RhoGEF TG macrophages. Previous reports showed that the RhoA pathway interference changes the morphology of M2 macrophage and inhibits the expression of M2-specific molecular markers, but not M1 morphology and M1-specific markers (18). The present study demonstrates that p190RhoGEF transgene over-expression changes the morphology of macrophages to be roundish as in M1 subtypes and inhibits the response of macrophages to an inflammatory condition. Thus, proper activation of RhoA might be required for the maintenance of the morphology and function of macrophages.

p190RhoGEF was identified as a specific GEF for RhoA activation, which is important for the regulation of the actin cytoskeleton (1, 14, 15, 28). The actin cytoskeleton is crucial to multiple cellular processes essential for an optimal immune response. Regulating the actin cytoskeleton through the small GTPase RhoA pathway has been shown to change macrophage shapes and functions (18, 19). Our data also show actin polymerization followed by a shape change in p190RhoGEF over-expressed TG macrophages. We used an inhibitor of Rho GTPase, rhosin, to determine whether the inhibition of RhoA would affect the shape and function of TG macrophages. The level of polymerized actin in the TG macrophages was reduced to that of the LTM macrophages by the rhosin treatment, and the shapes of the TG macrophages changed after that, spreading well, as seen with the LTM macrophages. Moreover, ROS and inflammatory cytokine production by the rhosin-treated TG macrophages in response to LPS also recovered to the level seen in the LTM macrophages. These results clearly demonstrate that the actin cytoskeletal change driven by the over-expression of p190RhoGEF changed the shape, and consequently the function, of macrophages.

Here, we have shown that the over-expression of p190RhoGEF changes the shape of macrophages and inhibits M1 polarization and function in an inflammatory situation. We have also shown that the inhibition of RhoA function in p190RhoGEF over-expressed TG macrophages removes the shape change and results in functional recovery. The present study thus suggests that morphological changes in macrophages caused by the actin cytoskeleton affect macrophage subset polarization and functions, although the detailed mechanisms remain to be studied. The results of this study provide new insights into the regulation of macrophages, which significantly affect innate immune responses during the pathogenesis of various inflammatory diseases.

## Data Availability Statement

The original contributions presented in the study are included in the article/supplementary material. Further inquiries can be directed to the corresponding author.

## Ethics Statement

The animal study was reviewed and approved by Ewha Institutional Animal Care and Use Committee.

## Author Contributions

SC and YA conceived and performed the experiments, analysed the data, and drafted the manuscript. EL and MY conducted ELISA and PCR experiments and analysed the results. JL participated in study design, data analysis, and writing of the final manuscript draft. All authors contributed to the article and approved the submitted version.

## Funding

This study was supported by National Research Foundation of Korea (NRF) grants funded by the Korean government (MSICT) (2012R1A5A1048236 and 2019R1A2C1007743 to JL).

## Conflict of Interest

The authors declare that the research was conducted in the absence of any commercial or financial relationships that could be construed as a potential conflict of interest.

## Publisher’s Note

All claims expressed in this article are solely those of the authors and do not necessarily represent those of their affiliated organizations, or those of the publisher, the editors and the reviewers. Any product that may be evaluated in this article, or claim that may be made by its manufacturer, is not guaranteed or endorsed by the publisher.
